# Keeping the Agenda Current: Evolution of Australian Lived Experience Mental Health Research Priorities

**DOI:** 10.3390/ijerph19138101

**Published:** 2022-07-01

**Authors:** Amelia Gulliver, Alyssa R. Morse, Michelle Banfield

**Affiliations:** Centre for Mental Health Research, National Centre for Epidemiology and Population Health, The Australian National University, Acton, ACT 2601, Australia; alyssa.morse@anu.edu.au

**Keywords:** mental health, research priorities, qualitative research, lived experience, consumers

## Abstract

The value of including consumers’ and carers’ views at the early stages of study design is increasingly being recognised as essential to improving the relevance and quality of research. One method of achieving this is by actively seeking and regularly updating consumer and carer priorities for mental health research. The current study presents priorities for mental health research collected from two virtual World Cafés with consumers and carers (*n* = 4, *n* = 7) held in 2021. Over 200 priorities were identified (13 themes, 64 subthemes), which were then compared with two combined data collection activities from 2013 (face-to-face forum; *n* = 25), and 2017 (online survey; *n* = 70). There appears to be some evolution in consumer and carer priorities over time. A key difference was that in the previous studies, mental health service issues were at the individual service delivery level, whereas in the current study, a broader focus was on mental health systems of care and issues around service funding, accessibility, and equity of access. It is possible these changes may also have resulted from key differences between the studies, including the methods, setting, and participants. Overall, similar to our previous studies no clear priorities were identified; however, a significant number of important research topics were identified by consumers and carers, providing a rich agenda from which to improve the management of mental health.

## 1. Introduction

The expertise of people with lived experience of mental health problems as consumers and carers is becoming increasingly recognised as a critical component in research [1,2]. The translation of basic scientific research into clinical practice has historically taken nearly two decades [3]. However, for translational research to be effective, the impact of the research through its clinical applications must be kept at the forefront [4]. New funding models for health and medical research are beginning to place a higher value on the real-world impact of work [5], with greater expectations in funding applications for researchers to be able to demonstrate the practical changes that have been made as a result of previous research. An important method of increasing research impact and enhancing the potential for the research to have relevance is ensuring that it meets the needs of people with lived experience or ‘consumers and carers’ [6].

The importance of the growing consumer and carer research field cannot be understated. Lived experience involvement in priority-setting research is advancing at a rapid pace, as indicated by research using the Roadmap for Mental Health Research in Europe (e.g., [7,8,9]) and the James Lind Alliance framework (e.g., [10,11,12]), which is adaptable across different types of consumer groups in healthcare [13]. For successful translation of research, it has been argued that stakeholder groups must work together [5]. However, priorities differ across stakeholder groups, with lived experience groups typically placing higher importance on clinical and applied research over basic science [4], and social interventions rather than biomedical ones [8]. Those with lived experience offer unique and important expertise, particularly in identifying overlooked [14] or cutting-edge, emerging issues [1]. Thus, priority-setting by consumer and carers groups is critical, ensuring the focus of the research remains on the end goal, improving mental health via policy and practice [4].

ACACIA: The Australian Capital Territory (ACT) Consumer and Carer Mental Health Research Unit, at The Australian National University (ANU), was established in 2013 with local government funding from ACT Health. The ACACIA research unit was created specifically to resource collaborative research that both partners with consumers and carers, and is led by them. Thus, ACACIA is purposely staffed by researchers with lived experience as a consumer or carer. The aim of the unit is to facilitate the engagement of people with lived experience in the community, into relevant, high-quality mental health research [15]. The research unit has also provided an effective bridge between mental health communities and academic groups [15]. A key objective of ACACIA is the collaborative development of a research agenda to address consumer- and carer-identified issues, such as service gaps in the ACT and Australia more broadly. Keeping these priorities up to date is also important to maintaining flexible and responsive research to both changing preferences and healthcare transformation [6].

ACACIA has undertaken two previous consumer and carer priority-setting studies to date. The original study comprised a half-day face-to-face forum held in Canberra, Australia in 2013 (*n* = 25), which used small focus group style discussions to generate and rank 79 research priorities specifically that were considered important to “*be the focus of research within the ACT*”, where the ACT is the Australian Capital Territory, a small region of Australia. This process was followed by a large group discussion and individual ranking of priorities listed on large sheets of paper using coloured dots [6]. We also asked how participants would like to engage in research [15]. Four years later in 2017, we updated this research by conducting an Australia-wide online survey (*n* = 70), to ensure the topics were relevant across the country, participants were asked to rate and rank the topics that were generated by the earlier study [6]. To keep our agenda current, the aim of the present study was to again update this work a further four years later in 2021, to renew priority-setting in mental health research for consumers and carers, whilst also providing information for a concurrent project to develop a National Disability Research Agenda. An in-depth discussion of the methods and the challenges encountered has been previously published [16].

### Aims

Our overall research question was: What do Australians with lived experience of mental health problems as either consumers or carers consider to be priorities for mental health research? The primary aim of this paper was to explore and analyse consumer and carer priorities for mental health research. A secondary aim was to compare the results with two previous studies [6] to examine how these priorities may have changed over time.

## 2. Materials and Methods

### 2.1. Participants and Recruitment

Participants self-identifying as mental health consumers and/or carers were invited to participate in one of two World Cafés. They were recruited via advertisements distributed to our networks of consumer and carer organisations nationally (e.g., ACT Mental Health Consumer Network, Carers Australia), on social media and the ACACIA research group webpage. Potential participants registered their interest in the study by contacting the researchers in response to the advertisement.

In our previous work [17], participants have emphasised the importance of confidentiality when discussing mental health issues. To assist with this in the current study, we chose not to collect or report personal demographic information.

### 2.2. Ethics and Consent Processes

Consistent with ethical standards [18], participants were provided with the study information sheet and asked to sign and return a consent form that confirmed they had read and understood the information sheet prior to participating, after which they were provided with a link to the World Café using the Zoom videoconferencing software (Zoom Video Communications Inc., San Jose, CA, USA). Though we did not use it, in the case that any potential participants had received the link (i.e., forwarded from a friend, etc.) but not yet completed consent, we had information and consent forms ready to conduct on-the-spot consent with support from a researcher. The information form explicitly informed participants of the potential risks of the discussion group. To protect potential participants, we also asked them not to sign up for the research, if they were currently experiencing mental health symptoms that were causing them distress. We also provided them with a list of support services attached to the information sheet and encouraged them to contact their preferred support person or one of the services on the sheet if required. Finally, whilst we did not expect any distress, we introduced participants to a registered psychologist, who had significant previous experience providing support for mental health both in person and over Zoom, who remained in the main Zoom room as part of the group to privately support any individuals who may have become distressed and refer them to support if needed. The psychologist support was not used by any of the participants.

### 2.3. Procedure

#### 2.3.1. Delivery Mode and Tools

Given the risks of COVID-19 transmission that applied during 2021 for in-person settings, we used an online format in place of face-to-face discussions, using Zoom video conferencing (Zoom Video Communications Inc., San Jose, CA, USA). We also used an online polling platform, Slido (sli.do s.r.o, Bratislava, Slovakia) in place of ranking exercises using in-person procedures, such as sticky dots and priority lists on paper. Three rounds of small group discussions were planned using the Zoom breakout room feature to create discussion “tables”, followed by one final large group discussion. Because the final recruitment (*n* = 11) fell short of the target (*n* = 40), in both groups, all participants instead remained in the main discussion room for all questions (see Section 2.4. Questions, below). This altered the method somewhat because there was no shuffling of group participants, the discussion became more like a virtual nominal group method, which typically progresses from a stage of silent idea generation, through to group discussion and then finally ranking of priorities via voting [19].

#### 2.3.2. World Café Method

World Cafés are a relatively recent method for facilitating group discussions [20]. They are highly useful for bringing large groups together to work more efficiently towards a single conversation [21]. To do this, small groups are formed where people are brought together in a comfortable context similar to a café, around a “table” to discuss a particular issue [22]. This can either happen physically or virtually in the case of the current study. The researchers then shuffle participants to new groups (or tables) to discuss a new issue, a process that is repeated for each question until all participants have contributed to all questions [22]. World Cafés have been successfully conducted online previously [23] demonstrating the effectiveness of this method, particularly in its ability to efficiently gather the views of people who may not be otherwise able to converse in-person, such as experts across the world [23]. Our recruitment goal was 40 participants. This was both to ensure we would have sufficient numbers of people to stimulate discussion around each virtual ‘table’, and to generate a greater number of topics (*n* = 79) than the original study did with 25 participants [6]). However, the large volume of information collected in the first two groups (201 topics) significantly exceeded our expectations based on the original study. In addition to this, and because of tight project timeframes, we chose not to conduct any further discussion groups.

Two 2.5-h virtual World Cafés were held during April 2021. The current study authors facilitated the discussions. To maintain confidentiality and maximise the participants’ comfort whilst discussing potentially sensitive issues, sessions were not recorded. Notes were taken by facilitators during discussions. Prior to each group, a facilitator read out a housekeeping script that explained how to use key Zoom features, reminded participants about voluntary participation and confidentiality, and described principles for the nature and content of discussions, including a request to specifically consider disability-related issues for the National Disability Research Agenda project, and described available support and how to seek it if needed.

### 2.4. Questions

The three discussion questions developed by the lived experience researchers (authors AG, MB, ARM) are listed below; the first two questions focused on priorities are discussed in the current paper. Question 3, which focuses on engagement with research will be discussed elsewhere, in the context of lived experience engagement practice (Banfield et al., forthcoming).

1. **What are the main issues you see as important in mental health in Australia?**
  *Prompts:* What are the issues or problems that are important to you or the people you support? Are there any potential ways that these issues could be improved for you, or for the people you support?
2. **What sort of research would you like to see prioritised in a national research agenda?**
  *Prompts:* What things do the government or other agencies need to know more about so they can better address your and your family’s needs? Is there a specific program, service, or treatment that you think should be evaluated? Is there a particular illness or group that we should focus on?
3. **How do you currently engage with research?**
  *Prompts:* What features of the research do you think make it useful to you or others? How do you find out about participating in mental health research? How would you like to be informed about how to help with being involved in conducting research? How would you like to engage with research in the future?

### 2.5. Ranking of Priorities

**Figure 1:**
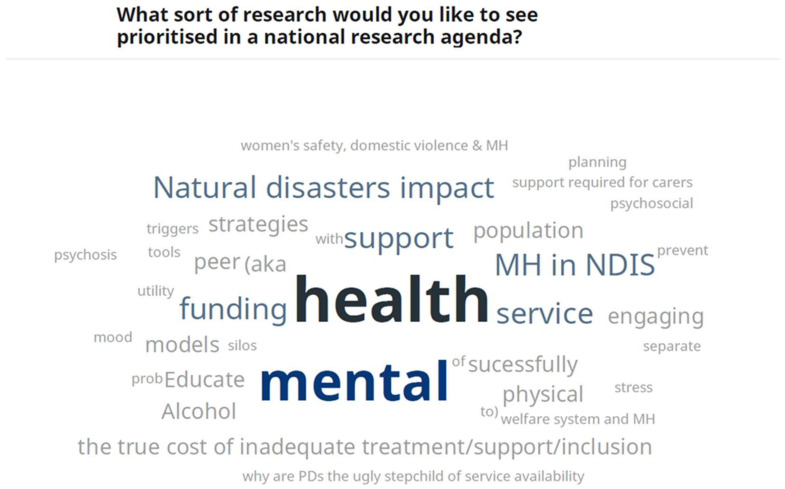
Slido poll for question 2 (Group 2; *n* = 7).

We used an online polling program called Slido to capture participants’ ideas and engage them in the discussions. During each session, participants anonymously entered words and phrases (“topics”) that they felt answered the research questions. Slido then automatically generated a visual “word cloud” that was updated for all participants and researchers in real-time. Figure 1 shows an example word cloud from question 2 (group 2; *n* = 7). Participants could emphasise topics by re-typing those already visible in the word cloud. To clarify topics and contextualise them, whilst viewing the cloud, facilitators encouraged further discussion of emphasised topics during each session. After the discussion of each of the three questions listed in Box 1, all participants took a 20-min break. During this break, the researchers downloaded the topics that had been entered into the Slido word clouds before copying and pasting them into two polls in Slido. In the final group discussion, participants clarified the topics raised in each of the three preceding discussions before voting on priority areas. To vote, they were asked to select one topic they thought was the *most important*, their highest priority topic, and the one topic they thought was the *least important*, their lowest priority topic.

### 2.6. Data Analysis

Our work follows a primarily constructivist paradigm, consistent with phenomenological qualitative research approaches [24]. We seek to explore and understand the perspectives of people with lived experience of mental health issues, personally or as a carer for someone who does. Written topics from questions 1 and 2 in the Slido poll were collated, and then inductive thematic analyses [25], consistent with our constructivist approach were conducted using NVivo 12 (QSR International) to assign topics to themes by one of two researchers (MB; AG). Author MB developed the initial coding frame from the issues raised in question 1, using open coding to generate codes, and constant comparative analysis to develop the structure across both groups [25]. Open codes were then organised into themes and subthemes to represent higher-level concepts. Consistent with our previous work, wherever possible, the language used by participants was preserved in theme and subtheme names. Author AG reviewed the structure and coded responses to question 2 against the initial frame, adding and refining codes as necessary to accommodate the different focus of question 2. The thematic structure was finalised by discussion between all three authors. Notes taken during the discussions were used to contextualise the theme list. The highest and lowest priority topics were also collated and tabulated. Finally, the agreed themes were compared with the previous studies by discussion between the three current study authors (AG, ARM, MB).

## 3. Results

A total of 11 people with lived experience as a consumer and/or carer, attended the World Cafés (*n* = 4, *n* = 7). During discussions, participants self-identified as a variety of genders and sexual orientations and ranging in age from young to older adult, but specific details of age and identification were not recorded. A brief summary of the discussions for each of the two questions is included below, to contextualise the written topics that were coded.

### 3.1. Current Study Results

For questions 1 (the main issues in mental health in Australia) and 2 (national research agenda priorities), overall, the first group (*n* = 4) generated 47 topics, and the second group (*n* = 7) 154 topics (total = 201 topics). We note this number differs from that listed in our paper describing the methods [16] as we located one additional item that was omitted in error during the transferring of the collated lists used for priority-setting. Initial coding for question 1 generated 22 themes and 39 subthemes. Eleven themes and subthemes were added to the coding structure to accommodate the responses to question 2 that did not fit into the structure created by the coding of question 1.

Table 1 presents the final coding structure developed by consensus between authors. Consistent with the scope of our previous work, the final set comprised 13 themes and 64 subthemes. Themes are ordered by approximately how much focus they received in both written responses and discussion, with subthemes placed in alphabetical order. Major areas are briefly described in the following sections, including specific topics that were raised in the verbal discussions.

#### 3.1.1. Main Issues for Mental Health in Australia

Written responses entered into Slido focused heavily on services and systems of care, particularly the funding issues for community-based services and programs, which were described as being only ever available short term due to a lack of funding. Conversation on these issues reflected a perception that federal government funding was primarily to support more severe impairment, and that community and mental health organisations were being left to pursue small or atypical funding options to stay afloat. Equity of access, and accessibility of services, especially acute care, were also seen as a significant problem within this theme, although others noted a need for alternatives to hospital and psychiatry-based services to offer more holistic and trauma-informed care. Psychological supports in prisons and forensic services were singled out as settings especially needing attention. In the verbal discussion, participants also raised issues around dual diagnosis, and comorbidity, and highlighted the importance of focusing on the whole of the person, noting that the siloing of services and overshadowing of physical health problems in people with mental health problems were counterproductive. Some discussion was had around the ‘missing middle’, where many services were felt to be available for mild and severe mental illness, but limited support for those with moderate symptoms.

Consistent with the request to consider issues specifically related to disability, the other major area of interest was psychosocial disability and particularly the National Disability Insurance Scheme (NDIS). The NDIS is a state and federal government funded support system for people with disability, their families and their carers [26]. In the verbal discussions, participants described the NDIS as being tricky to navigate and having difficulties engaging with the scheme as a mental health consumer, due to a reported lack of appropriate skills or experience amongst NDIS staff. The current setup of the scheme was not seen as fit-for-purpose for mental health problems, with written topics noting problems with assessments, episodic care needs and sectoral differences between the disability and mental health sectors. The discussions on these areas particularly noted that the episodic nature of mental illness meant that people could fluctuate in their ability to qualify for NDIS support and to receive needed services, compounded by tensions between the focus on disability for the NDIS versus recovery for the mental health sector. Alongside these issues specifically for those with a disability, was a broader discussion of inclusion and support, particularly the need to focus on social inclusion and the reintegration of people experiencing mental health issues into the community and workplaces.

Carer issues, and in particular the role of carers and families in supporting people with mental illness, were of interest to a number of participants. Participants discussed the lack of recognition of the impact on carers, with a number of points raised including the lack of access to treatment and support other than psychiatry, challenges around privacy for consumers and carers, and a suggestion that service providers need to seek and recognise carer input.

Individual themes that received several mentions included stigma and discrimination, in the media and the general public, with a call to “*normalizing the conversation*” around mental health and facilitating better social inclusion. The context of natural disasters was also raised, with participants noting COVID-19, the recent bushfires, floods, and the general impact of natural disasters in Australia on mental health.

#### 3.1.2. Priorities for Research in a National Agenda

When asked to consider mental health issues in the context of priorities for research, there were both similarities and differences in the topics discussed compared with the first question. As for question 1, priorities within the major theme on services and system issues again centred on the accessibility of services, alternatives to hospitals, and holistic “whole of person” care. However, a new topic was introduced, regarding how and where government funding was spent. Verbal discussions on this topic concerned the issue of “*cost-shifting*” between state and federal funding responsibilities. Participants were interested in how to address these funding issues to improve the coordination of services at a national level, reduce the fragmentation of services, and the lack of integration between the NDIS and other services, including in the general disability space. Housing and welfare issues and the need to reduce incarceration in the justice system were also added to the system issues identified as being important for future research.

The NDIS, particularly addressing the episodic nature of mental health problems was again mentioned as being important for research, as well as the impact on carers, including how carers contribute, i.e., the “*national economic & social impact of carers*”.

The separation of physical and mental health was also raised as an important topic for research, where the current distinction between the two in relation to services was questioned—“*when did the brain stop being a bodily organ?*” Other important causes and risk factors for mental illness, such as ageing and domestic violence were also introduced at this point in the discussion. Further areas of emphasis included medication issues, and specific mental disorders, including schizophrenia and related disorders.

Table 2 presents the participants’ perceived highest and lowest priority items as selected from the full combined list of all responses to questions 1 (main issues) and 2 (research priorities). All participants selected different priorities; thus, no priorities were identified as the highest or lowest priority by more than one participant. However, the highest priorities broadly reflected the amount of support for each priority in the written response coding, and the verbal discussions, focused on holistic, community-based services, especially for those currently missing out, inclusion and support, and prevention. The lowest priorities included various topics on stigma and perceptions, the influence of social media and some specifics on treatments. Some topic areas appeared in both lists, including the specific topic of increasing the evidence base for peer support, funding issues and interest in personality disorders.

### 3.2. Comparison with Previous Priority-Setting

To explore the progression of consumer and carer priorities during the 8 years encompassing the three processes, the lists of themes and subthemes developed for the current study were compared with the thematic areas and topics developed in the previous two. There was continuity in analysis, with author MB being involved in the analysis for all three studies, ARM conducting analyses for the 2017 study [6], and ARM and AG assisting with the current study analysis. Table 3 presents the comparison at the thematic level; the full list of themes and topics from the original study [6] is reproduced in Supplementary File S1.

At the thematic level, there were broad similarities in the major themes, especially in the largest areas on services, disability and carer issues. Only one theme from the current study, *prevention and early intervention*, did not feature at all in the previous thematic areas, but there were a number of current themes that were only represented as individual topics in the earlier work, including *risk factors*, *specific populations* and *recovery.* In addition, there were three substantial thematic areas in the earlier work that did not feature in the current study and a further three that were only represented at the subtheme level with a slightly different focus. There was no discussion in the current study that specifically focused on *experiences of care*, *language* and *communication* or *legislation*. In addition, discussions on *health professionals*, *justice* and *comorbidity and physical health* were all in the context of service design and delivery (See Table 1 theme: service and system issues).

At the individual subtheme and topic level, further differences emerged. In the work conducted in 2013 and 2017, service issues were primarily at the individual service delivery level, looking at service pathways, communication within service settings and impact on recovery (See Supplementary File S1). Table 1 shows that in the current study there was a much greater focus on the mental health *system*, in particular, the influence of funding models and politics, the need to shift away from psychiatric and acute care, and to stop people “falling through the cracks.” Trauma-informed care and the influence of the public versus private systems appeared in both lists.

There were striking differences in the scope and nature of subthemes and topics for the psychosocial disability and peer workforce themes. In the 2013 study, conducted when both of these initiatives were still in formative stages, topics reflected the need to understand and define the issues and how they would integrate into the Australian mental health system. These formative issues were subsequently rated as some of the highest priorities in the survey study conducted in 2017. In the current study, discussions focused on the problems for the implementation of the NDIS, and the maturation of a peer support work into a true workforce. The lived experience involvement theme similarly evolved from a focus on how to expand involvement and improve tokenism to a human rights-based discussion on involvement at all levels of the system and types of research.

There were also substantial differences in the scope of the discussions on stigma and treatment and their relative importance as priorities. In the earlier work, stigma was an extensive thematic area, including how it impacted service delivery, involvement and outcomes. Likewise, treatment was a broad area developed in the 2013 study, covering specific therapies, care planning and clinical management. Both themes contained topics that were ranked in the top ten priorities for people with lived experience as both consumers and carers in the 2017 survey. However, in the current study, stigma and discrimination was only a minor theme and was included in some people’s lowest priorities, and treatment was restricted to optimising medications (part of an entire theme in earlier work), the role of physical activity and interest in specialised treatments, such as Eye Movement Desensitisation and Reprocessing (EMDR).

## 4. Discussion

This study provides an updated picture of the most salient issues in the Australian mental health system, the current research topics of interest and importance to Australian consumers and carers, and an indication of how they may be changing over time. Over 200 potential priority research topics were identified and categorised into a total of 13 major themes, and 64 subthemes. Mental health services and systems of care were a key focus for participants, with particular attention paid to issues around service funding, accessibility and equity of access. In line with the study’s goal to inform a National Disability Agenda, the NDIS was also an important area for issues and research topics, including the poor fit of the disability insurance scheme for mental health consumers, and its lack of integration with other services. The social and economic role of carers, and the recognition of the impacts they experience, were also topics of interest. No clear consensus was reached on the most important research priorities, with each participant selecting a different highest and lowest priority for research. However, the selected top-priority items reflected the focus on holistic, community-based, and accessible services observed in the data. These findings align with previous work, where lived experience priorities for research have primarily focused on applied research and holistic interventions beyond biomedical treatment [4,8]. Although some basic research (e.g., causes and risk factors) and biomedical-related research topics (e.g., optimising medications) were suggested.

Lived experience priorities for Australian mental health research appear to have shifted over time. These changes may be reflective of an evolving mental health care system, highlighting the value of regularly gathering lived experience perspectives to identify cutting edge and emerging issues [1]. For example, the 2013, 2017 [6,15] and current priority-setting studies captured key time points in the implementation of the NDIS, from the planning phase to the trial and roll-out of the service, and finally several years post-implementation [26]. The research topics developed by participants follow this development, from an original focus on formative issues (e.g., how is psychosocial disability defined in the NDIS, and how will it impact consumers and carers in Australia) to the current interest in improving how the service is implemented (e.g., psychosocial assessment, psychosocial disability left out). These findings demonstrate the importance of lived experience perspectives for identifying current areas of need, where research can be targeted with the best potential for real-world impact [1,13].

The scope of priority-setting discussions may also influence the kinds of research topics identified. For example, mental health services were a key area of focus across our three priority-setting exercises. At the 2013 forum, participants were asked to identify topics that they “think should be the focus of research within the ACT”, which is the Australian Capital Territory, a relatively small region of Australia [6]. This may have encouraged discussion around the individual-level impacts of health services; changes that could be made to improve local health service delivery and community experiences. The current study asked participants to consider research priorities on a national scale. This may have, in part, guided participants to focus on health systems issues (e.g., the allocation of government funding) with the potential to have an impact across Australia. However, differences in discussions between the studies may also be due to variation between participants, or changes in the mental health sector over time. Lived experience priority-setting ensures that the focus of research is to improve mental health care through policy and practice [4]. Our findings suggest that it is important to clearly define the scope of research priority-setting exercises, supporting participants to identify the most relevant and appropriate areas of need.

The way discussion questions are framed may also impact the kinds of research topics and priorities developed. In the current study, there was some overlap between the main issues identified as being important to mental health in Australia, and the research topics that participants wanted to see prioritised in a national research agenda. However, there were also some interesting differences. Many of the topics raised as being important in Australian mental health were not subsequently suggested as important topics for prioritising. There were also a small number of new topics that only emerged in the context of specifically discussing research priorities, such as ageing and the brain (neuroplasticity and functional ability); domestic violence and issues with the justice system; measurement issues, such as how mental health scales are being used for service delivery and funding issues, such as cost-shifting between state and federal government. This suggests that there can be clear differences between asking consumers and carers about important issues affecting people in general and asking them specifically about what is important to research. However, these differences may also have been due to the methods of the present study (e.g., the order of discussion questions); thus, it may be important to consider the types of discussion questions used when planning a priority-setting exercise.

### Limitations

The current study has several limitations to consider. Given constraints relating to deadlines for contribution to an external project, we were only able to conduct two groups (*n* = 11), and our final numbers fell well short of planned (*n* = 40). Whilst the views of the participants may not be representative of all consumers and carers, we note that they generated a large number and variety of priorities for research. An additional limitation is that because of the low number of participants, we had to deviate from our plan of conducting rotating discussions in a World Café method, and instead used nominal group methods [19,27]. However, given we also did not gain agreement on the participants’ highest or lowest priorities, we also did not technically conduct a consensus study. This may have been partly because of the final limitation, which was that our ranking activity likely had far too many priorities to rank to achieve a consensus. By forcing participants to select just one of the more than 45 (Group 1) or 150 (Group 2) topics, only 11 ideas could be endorsed as most important, and given the diversity in consumer and carer views, it was highly unlikely that in a small group we would gain more than one or two votes for any single priority.

## 5. Conclusions

People with lived experience are essential to identifying key areas of need in the mental health system and targets for future research. Consumers and carers can be prolific generators of ideas and are able to differentiate between issues in mental health in Australia, and topics that are important future areas of research. Continuous checking in of lived experience priorities for research ensures that research has practical impact and remains focused on those it fundamentally aims to support. In addition, working in partnership with consumers and carers enables research to be responsive to the current needs of consumers and carers, and the evolution of these needs over time. No clear priorities were identified. However, the current study has generated a significant number of topics that consumers and carers identified as important for future research, providing a foundation for the development of lived-experience-informed contemporary research agendas to improve mental health.

## Figures and Tables

**Table 1:** Coded themes for questions (1) main issues, and (2) research priorities for mental health in Australia.

Themes and Subthemes
**Service and system issues**
Accessibility, e.g., community supports, costs
Acute care, lack of beds
Alternatives to hospital, availability of appropriate services
Alternatives to psychiatry, holistic treatment
Awareness of services
Diagnostic overshadowing
Evaluation of programs
Falling through cracks
Implementation of plans, services, inquiries
Lack of funding, esp recurrent, short programs
Least restrictive practice
* Measurement issues
Missing middle
Psych support in prisons, forensic services, alternatives
Public private split
Rural and remote mental health, services
Staffing capacity and capability
Trauma-informed care
* Welfare and housing
Policy and political impact
Funding relative to physical health
Cost shifting Federal/State
* Costs to the individual
Police and MH services
* Justice system
**Psychosocial disability**
Balance of illness and independence
Disability sector vs MH sector
Functional ability
NDIS—episodic care, independent assessments, peer services
Psychosocial assessment
Psychosocial disability left out
**Inclusion and supports**
Normalising workplace reasonable adjustment
Seeking work, homelessness
Social inclusion, reintegration
Supported accommodation
**Carers**
Carer peer support
Carer roles and impacts, families
**Causes and risk factors**
* Ageing
Disasters
* Brain research
* Domestic violence
Social media
**Disorder specific**
Best practice personality disorders
Dual diagnosis
Eating disorders
Neurodiversity and MH
* Psychosis, schizophrenia, schizoaffective disorder
**Prevention and early intervention**
Early intervention
Mental health in schools
Resilience
Youth supports, prevention
Suicide prevention
**Specific populations**
Multicultural support
LGBTIQ+ identity, access and inclusion
Aboriginal and Torres Strait Islanders
**Lived experience involvement**
* Involvement in quantitative research
Lived experience in policymaking
Consumer rights
**Treatments and other interventions**
Optimising medications
Physical activity
* Specific treatments, e.g., EMDR
**Peer workforce issues**
Peer support and workforce
Peer support in industry (e.g., mates in construction)
**Stigma, discrimination and associated behaviours**
Perceptions of mental health as separate to health
**Recovery**

***Note:*** The coding set was generated by coding question 1 first. * Themes and sub-themes that were new, and generated by coding the responses to question 2 (research priorities); EMDR: Eye Movement Desensitisation and Reprocessing, MH: Mental Health, NDIS: National Disability Insurance Scheme.

**Table 2:** Highest and lowest priorities as voted by participants (*n* = 11).

Highest	Lowest
Better access to community support when needed	Co-designing information about medications
Creative ways to increase funding to increase research and services	COVID
dementia and older people	Forget reducing Stigma and look at addressing behaviour emanating from that attitude
* greater peer support evidence base	government funding
How to educate the population in (trying to) prevent Mental Illness	* greater peer support evidence base
medical research negative symptoms of schizophrenia	perceptions of ‘mental’ health
missing middle	Personality disorder best practice
more holistic/‘whole-of-person’ treatment	social media—increasing anxiety
reasonable adjustments—what are they, who decides, seeing more	stigma
Re-integration into community	TMS available in multiple areas and regional
why are PDs the ugly stepchild of service availability	--

***Note:*** Direct topic quotes presented in alphabetical order. * Denotes topic that appeared in both lists; --One participant did not provide a lowest priority; PD: Personality Disorder, TMS: Transcranial Magnetic Stimulation.

**Table 3:** Thematic areas from current and previous research in Banfield et al. [6].

Thematic Areas from Current Research	Thematic Areas from Banfield et al. [6].
Service and system issues	Services
Psychosocial disability	National Disability Insurance Scheme
Inclusion and supports	Not a separate theme, but individual topics in ungrouped “other”
Carers	Carers, families and friends
Causes and risk factors	Not a separate theme, but an individual topic in ungrouped “other”
Disorder specific	Not a separate theme, but personality disorders in stigma
**Prevention and early intervention**	* Not featured
Specific populations	Not a separate theme, but individual topics in ungrouped “other”
Lived experience involvement	Consumer and carer involvement
Treatments and other interventions	Treatment Medications
Peer workforce issues	Peer to peer
Stigma, discrimination and associated behaviours	Stigma
Recovery	Not a separate theme, but individual topic in ungrouped “other”
Not a separate theme, but some aspects in service and system issues	Comorbidity and physical health
* Not featured	**Experiences of care**
Not a separate theme, but similar subtheme in service and system issues	Health professionals
Not a separate theme, but similar subtheme in service and system issues	Justice
* Not featured	**Language and communication**
* Not featured	**Legislation**

***Note:*** * Not featured in the identified themes.

## Supplementary Materials

The following supporting information can be downloaded at: https://www.mdpi.com/article/10.3390/ijerph19138101/s1, Supplementary File S1: Research priority ratings for consumers, carers and consumer/carers from the original study.
