# Supplementary material for: Keeping the Agenda Current: Evolution of Australian Lived Experience Mental Health Research Priorities
Source: Int J Environ Res Public Health. 2022 Jul 1;19(13):8101. doi: 10.3390/ijerph19138101 (PMC9265903; doi:10.3390/ijerph19138101)
Supplement: Supplementary file 1 [file ijerph-19-08101-s001.zip › ijerph-1767277-supplementary.pdf]

Supplementary file S1. Research priority ratings for consumers, carers and consumer/carers from the original study.

Research Topic	Topic Area	Priority ranking (Percentage important ratings)		
		Consumer	Carer	Consumer/ Carer
How to implement internationally recognised models of peer support in Australia	*Peer to Peer*	1 (94)	24 (70)	72.5 (44)
Over-representation of mental illness in the justice system	*Justice*	2 (88)	1.5 (100)	11 (78)
How is psychosocial disability defined in the NDIS, and how will it impact consumers and carers in Australia?	*National Disability Insurance Scheme*	4.5 (88)	9 (80)	15.5 (76)
How does the use of language include/exclude individuals?	*Language and Communication*	4.5 (88)	69 (44)	55.5 (56)
Consumers' experiences of peer to peer services	*Peer to Peer*	4.5 (88)	39 (60)	64.5 (50)
Social inclusion	*Other*	4.5 (88)	15.5 (78)	44.5 (60)
How participation works in practice (tokenism vs. real involvement)	*Consumer & Carer Involvement*	7.5 (85)	24 (70)	11 (78)
What is helpful in recovery-oriented services?	*Experiences of Care*	7.5 (85)	9 (80)	32 (65)
Peer-led services - What are the gaps? (e.g. support groups)	*Peer to Peer*	10 (84)	46.5 (56)	55.5 (56)
How to recruit and train peer workers - What is going on, and where? Where is it embedded? How are they being supported?	*Peer to Peer*	10 (84)	39 (60)	55.5 (56)
Mental health in LGBTIQ+ populations	*Other*	10 (84)	46.5 (56)	38.5 (63)

**Research Topic**	**Topic Area**	**Priority ranking (Percentage important ratings)**		
		**Consumer**	**Carer**	**Consumer/ Carer**
How is the consumer and carer voice integrated into policy and services? How are their contributions valued, and what indicators exist to demonstrate how their voice is used?	*Services*	12 (84)	52.5 (55)	21.5 (71)
How do we expand who is involved? (e.g. young people)	*Consumer & Carer Involvement*	13 (82)	15.5 (78)	64.5 (50)
Is medication what we want? Side effects, health impacts, alternatives, efficacy, cost-effectiveness	*Medication*	14 (79)	34 (64)	59 (55)
Stigma by health providers (mental health and others) - What do they believe and how does it impact?	*Stigma*	15.5 (79)	24 (70)	5 (83)
Are consumers being consulted about their experiences of care?	*Experiences of Care*	15.5 (79)	24 (70)	21.5 (71)
Impact of service delivery on consumers and carers - What contributes to recovery?	*Services*	17 (78)	58 (50)	7 (81)
What programs/supports can be devised for reaching individuals that are outside of NDIS scope?	*National Disability Insurance Scheme*	20.5 (78)	9 (80)	15.5 (76)
Peer support in public mental health system	*Peer to Peer*	20.5 (78)	46.5 (56)	24.5 (69)
Accommodation	*Other*	20.5 (78)	46.5 (56)	25.5 (69)
Employment	*Other*	20.5 (78)	5 (89)	7 (81)
Support in education settings	*Other*	20.5 (78)	15.5 (78)	77 (38)

**Research Topic**	**Topic Area**	**Priority ranking (Percentage important ratings)**		
		**Consumer**	**Carer**	**Consumer/ Carer**
Culturally and linguistically diverse perspectives within mainstream mental health system	*Other*	20.5 (78)	69 (44)	32 (65)
How do individuals adapt to changes in medication that impact lifestyle and quality of life?	*Medication*	24 (77)	52.5 (55)	38.5 (63)
Training of psychologists - How can consumer perspectives be incorporated?	*Health Professionals*	26.5 (76)	62.5 (45)	44 .5 (60)
Where do physical health concerns fit into health services when you have mental health problems as a main focus?	*Comorbidity & Physical Health*	26.5 (76)	39 (60)	11 (78)
Discrimination	*Justice*	26.5 (76)	15.5 (78)	27.5 (67)
How have people who have experienced trauma been cared for?	*Experiences of Care*	26.5 (76)	39 (60)	21.5 (71)
Trauma informed care - Why is it important, and how is it integrated into service delivery?	*Services*	29.5 (76)	39 (60)	42 (62)
Reach – Are services reaching the people that need them?	*Services*	29.5 (76)	3 (91)	27.5 (67)
Capacity for decision making/change in legislation and its application; consumer and carer experiences of this. What information is provided about legislation? What support is provided e.g. legal?	*Legislation*	32.5 (75)	15.5 (78)	47.5 (59)
To what extent do we follow human rights legislation on mental illness?	*Legislation*	32.5 (75)	24 (70)	15.5 (76)

**Research Topic**	**Topic Area**	**Priority ranking (Percentage important ratings)**		
		**Consumer**	**Carer**	**Consumer/ Carer**
Recovery and fulfilling potential	*Other*	32.5 (75)	15.5 (78)	64.5 (50)
Suicide: continuous care and support	*Other*	32.5 (75)	5 (89)	7 (81)
Is care traumatising?	*Experiences of Care*	35 (74)	9 (80)	15.5 (76)
Consumer and carer journey through service pathways - What works and what doesn't? What do clinicians think?	*Services*	36.5 (73)	34 (64)	52 (57)
Care coordination between mental health and physical health	*Comorbidity & Physical Health*	36.5 (73)	58 (50)	1 (89)
Consumer perspectives on use of labels - Which terms are useful/helpful, which are not?	*Language and Communication*	38.5 (72)	69 (44)	47.5 (59)
Mental health in culturally and linguistically diverse populations	*Other*	38.5 (72)	58 (50)	32 (65)
Children of people with mental illness	*Other*	40 (71)	29.5 (67)	38.5 (63)
Awareness and role of General Practitioners (GPs) - e.g., engagement with carers, language and communication skills with consumers and carers	*Services*	41.5 (70)	62.5 (46)	15.5 (76)
Exhaustion & burnout of mental health professionals - Impact on service support and delivery	*Health Professionals*	41.5 (70)	34 (64)	32 (65)
How many people with mental illness/disability are eligible for NDIS support?	*National Disability Insurance Scheme*	44.5 (69)	46.5 (56)	60 (53)

Research Topic	Topic Area	Priority ranking (Percentage important ratings)		
		Consumer	Carer	Consumer/ Carer
What forms of communication work for consumers and carers? (e.g. older people - less technology familiarity; younger people - social media, smart phones)	*Language and Communication*	44.5 (69)	84 (30)	19 (75)
Care planning - What makes a good mental health plan? (e.g. individualised, including perspectives of consumers, carers and clinicians)	*Treatment*	44.5 (69)	34 (64)	3.5 (84)
Transparency of clinical management - How does it respond and interact with consumers and carers?	*Treatment*	44.5 (69)	58 (50)	3.5 (84)
What are the experiences of and needs of people coming off medication? How are they being supported?	*Medication*	47 (68)	62.5 (45)	9 (80)
How mental health-aware are General Practitioners (GPs)?	*Health Professionals*	49.5 (67)	34 (64)	38.5 (63)
What is the role of a General Practioner (GP) -perceived and actual) - as part of the therapeutic alliance in care of mental health consumers?	*Health Professionals*	49.5 (67)	52.5 (55)	26 (68)
Stigma around Borderline Personality Disorder	*Stigma*	49.5 (67)	81 (33)	76 (39)
Stigma as a barrier to consumer involvement	*Stigma*	49.5 (67)	46.5 (56)	43 (61)
Alternative treatments - What are they, and how can they contribute to recovery? Holistic approaches, meditation, exercise	*Treatment*	52.5 (66)	76.5 (36)	75 (40)

Research Topic	Topic Area	Priority ranking (Percentage important ratings)		
		**Consumer**	**Carer**	**Consumer/ Carer**
Learned helplessness (experience with services)	*Other*	52.5 (66)	69 (44)	55.5 (56)
Consumers & Carers - Who is involved?	*Consumer & Carer Involvement*	55 (64)	15.5 (78)	55.5 (56)
Does the stigma in the mental health system worsen outcomes?	*Stigma*	55 (64)	29.5 (67)	32 (65)
What is the effect of caring?	*Carers, Family and Friends*	55 (64)	5 (89)	15.5 (76)
What sources of information do consumers and carers have faith in?	*Language and Communication*	57 (63)	81 (33)	64.5 (50)
How can medications be tailored to the individual?	*Medication*	58.5 (62)	62.5 (45)	50.5 (58)
Criteria for prescribing medications	*Medication*	58.5 (62)	52.5 (55)	38.5 (63)
What are clinician views on peer support?	*Peer to Peer*	60.5 (61)	81 (33)	72.5 (44)
Risk factors for mental illness	*Other*	60.5 (61)	46.5 (56)	32 (65)
How can we get mental health and other health professionals to work together more efficiently?	*Health Professionals*	63.5 (61)	20 (73)	50.5 (58)
Comorbidities and stigma	*Stigma*	63.5 (61)	69 (44)	64.5 (50)
What changes do people make in their own lives as a result of stigma?	*Stigma*	63.5 (61)	46.5 (56)	55.5 (56)
Who are the carers and what are they doing?	*Carers, Family and Friends*	63.5 (61)	24 (70)	47.5 (59)

Research Topic	Topic Area	Priority ranking (Percentage important ratings)		
		Consumer	Carer	Consumer/ Carer
Translation of clinical frameworks and guidelines into practice - Why is there a disconnect?	*Treatment*	66 (60)	52.5 (55)	38.5 (63)
Carers & bereavement – Are we offering enough counselling? Is it timely enough? Should it be offered in prisons?	*Carers, Family and Friends*	67.5 (59)	9 (80)	32 (65)
What is a peer?	*Peer to Peer*	67.5 (59)	81 (33)	86 (19)
What support is available when pain is a comorbid condition? How are people experiencing that?	*Comorbidity & Physical Health*	70.5 (58)	85 (22)	64.5 (50)
What is the evidence base linking mental illness with alcohol and other drugs?	*Comorbidity & Physical Health*	70.5 (58)	29.5 (67)	64.5 (50)
What kind of support would carers like?	*Carers, Family and Friends*	70.5 (58)	1.5 (100)	21.5 (71)
Is there such a thing as carer recovery?	*Carers, Family and Friends*	70.5 (58)	15.5 (78)	2 (88)
How do current protocols support consumer and carer journeys to recovery?	*Treatment*	73.5 (57)	24 (70)	69 (47)
How are Partners in Recovery (PiR), Personal Helpers and Mentors (PHaMs), support and clinical management working together?	*Services*	73.5 (57)	76.5 (36)	74 (43)
Bullying	*Other*	75 (56)	81 (33)	72.5 (44)
Trial of primary health care nurse within mental health teams – Does it improve physical health outcomes?	*Comorbidity & Physical Health*	76.5 (55)	74 (40)	84 (28)

Research Topic	Topic Area	Priority ranking (Percentage important ratings)		
		Consumer	Carer	Consumer/ Carer
Analysis of stigma according to disorder	*Stigma*	76.5 (55)	69 (44)	47.5 (59)
Bereavement	*Other*	78 (53)	69 (44)	82 (31)
How is 'privacy' interpreted by health professionals, and does it differ from consumer and carer interpretations?	*Health Professionals*	79.5 (52)	52.5 (55)	81 (32)
Stereotype formation	*Stigma*	79.5 (52)	58 (50)	79.5 (33)
Insurance	*Other*	81 (50)	29.5 (67)	85 (25)
Gender specific effects of medication	*Medication*	82 (47)	87 (18)	78 (35)
Effects of drug and alcohol use early in life	*Comorbidity & Physical Health*	83 (39)	69 (44)	72.5 (44)
Electroconvulsive therapy (ECT) - What information is given, does it follow best practice, what are consumers' experiences?	*Treatment*	84.5 (31)	86 (20)	83 (30)
Smoking cessation	*Other*	85.5 (31)	69 (44)	87 (7)
Do the public and private sectors work together? Consumer and carer experiences	*Services*	86 (30)	76.5 (36)	79.5 (33)

Note: Items are ranked in descending order of consumer importance rating percentages [from 1 (most frequently rated as "important") to 87 (least frequently rated as "important")]. The percentage rankings for the carer and consumer/carer participant groups are provided for comparison. Items sharing the same importance rating percentage were assigned a mean rank.
